# Supplementary material for: Complexome profiling of the Chlamydomonas psb28 mutant reveals TEF5 as an early PSII assembly factor
Source: Plant Cell. 2025 Mar 17;37(6):koaf055. doi: 10.1093/plcell/koaf055 (PMC12164590; doi:10.1093/plcell/koaf055)
Supplement: koaf055_Supplementary_Data [file koaf055_supplementary_data.zip › psb28_interactive.zip!/plots/intensity/qualityPlots/peptides/_Cre13.g571700.t1.1.html]

### Cre13.g571700.t1.1

Trivial name:   
SeqCoverage [median]: 9.90  
Mass: 56.26 kDa  
  
 

| Sequence | MissedCleavage | Mass [Da] | wtIntensity | muIntensity | Score |
| --- | --- | --- | --- | --- | --- |
| DLKPENILLSDKTQHAILK | 2 | 2175.23 | 0.00 | 862.54 | 26.81 |
| LALVVLAR | 0 | 853.57 | 862.34 | 1117.81 | 88.73 |
| TLTDNDVKR | 1 | 1060.55 | 315.53 | 343.70 | 62.46 |
|  |  |  | 1177.9 | 2324.0 |  |
